# Antioxidant *Baccharis trimera* Leaf Extract Suppresses Lipid Accumulation in *C. elegans* Dependent on Transcription Factor NHR-49

**DOI:** 10.3390/antiox11101913

**Published:** 2022-09-27

**Authors:** Flávia Roberta Monteiro Souza, Giovanna Melo Martins Silva, Cesar Orlando Muñoz Cadavid, Lucas dos Santos Lisboa, Maylla Maria Correia Leite Silva, Weslley Souza Paiva, Marcelo José Pena Ferreira, Riva de Paula Oliveira, Hugo Alexandre Oliveira Rocha

**Affiliations:** 1 Laboratório de Biotecnologia de Polímeros Naturais (BIOPOL), Programa de Pós-graduação em Bioquímica e Biologia Molecular, Centro de Biociências, Federal University of Rio Grande do Norte—UFRN, Natal 59078-970, Brazil; 2 Laboratório de Genética Bioquímica (LGB), Programa de Pós-graduação em Biotecnologia, Centro de Biociências, Federal University of Rio Grande do Norte—UFRN, Natal 59078-970, Brazil; 3 Laboratório de Fitoquímica, Departamento de Botânica, Instituto de Biociências, Universidade de São Paulo—USP, Rua do Matão, 277, São Paulo 05508-090, Brazil

**Keywords:** carqueja, asteraceae, phenolic compounds, obesity, antioxidant, *Caenorhabditis elegans*

## Abstract

Obesity is a global public health problem that is associated with oxidative stress. One of the strategies for the treatment of obesity is the use of drugs; however, these are expensive and have numerous side effects. Therefore, the search for new alternatives is necessary. *Baccharis trimera* is used in Brazilian folk medicine for the treatment of obesity. Here, *B. trimera* leaf extract (BT) showed antioxidant activity in seven in vitro tests, and it was not toxic to 3T3 murine fibroblasts or *Caenorhabditis elegans*. Furthermore, BT reduces the intracellular amount of reactive oxygen species and increases *C. elegans* survival. Moreover, these effects were not dependent on transcription factors. The inhibition of fat accumulation by BT in the *C. elegans* model was also investigated. BT reduced lipid accumulation in animals fed diets without or with high amount of glucose. Furthermore, it was observed using RNA interference (iRNA) that BT depends on the transcription factor NHR-49 to exert its effect. Phytochemical analysis of BT revealed rutin, hyperoside, and 5-caffeoylquinic acid as the main BT components. Thus, these data demonstrate that BT has antioxidant and anti-obesity effects. However, further studies should be conducted to understand the mechanisms involved in its action.

## 1. Introduction

Obesity is a chronic, non-communicable disease characterized by an excessive accumulation of body fat. It is a major global epidemic, representing countless losses to society and public health systems as it is associated with significant morbidity and mortality. According to the World Health Organization (WHO), the worldwide prevalence of obesity almost tripled between 1975 and 2016 [1]. The estimate for 2030 is that 18% of the adult population will be obese [2]. Obesity is associated with several diseases such as hypertension [3], dyslipidemia [4], some types of cancer [5], cardiovascular diseases [6], and type 2 diabetes mellitus [7].

People who followed a diet (500 kcal/day less than that required for weight maintenance) rich in carbohydrates or protein for six months showed weight loss regardless of the diet. This was associated with decreased oxidative stress [8]. In fact, studies have reported the role of oxidative stress in the pathogenesis of obesity and its associated risk factors [9,10,11,12]. Oxidative stress is caused by the imbalance between the formation of reactive species and the ways to neutralize these species and/or the damage caused by them. In fact, reactive species, mainly oxygen reactive species (ROS), can regulate mitochondrial activity, and can stimulate adipogenesis and lipogenesis, including stimulating the differentiation of preadipocytes into mature adipocytes, thereby increasing the size and number of adipocytes. Moreover, they play an important role as agents that regulate the energy balance in hypothalamic neurons that control appetite [13].

The therapeutic approach for weight gain and obesity is based on lifestyle modification, prevention programs, behavioral modification, and in some cases, the use of medications or bariatric surgery [14,15]. Drugs used in the treatment of obesity are expensive and have harmful side effects such as nausea and vomiting, elevation in blood pressure and heart rate, fecal urgency, oily stools (steatorrhea), and reduced absorption of fat-soluble vitamins [16]. Therefore, among the efforts to circumvent these problems, other options for the treatment of obesity, such as natural products, are being evaluated [17].

Natural antioxidants have recently gained attention due their capacity to counteract the deleterious effects of oxidative stress and diseases associated with it. Their mechanisms of action include antioxidant properties, such as metal chelating, free radicals’ scavenger, and changes in gene transcription through the induction or inhibition of transcription factors [18]. Another cytoprotective mechanism, used by natural compounds against excess ROS, is the control of the signaling pathway of the nuclear transcription factor erythroid 2p45 (NF-E2)–related factor 2 (Nrf2) [19], and within this context are medicinal plants, which are rich sources of antioxidant compounds [20].

Medicinal plants are increasingly studied sources due to their low cost, high availability, and safety profile [21]. The activities of medicinal plants are attributed to the phytochemicals produced by these species, including flavonoids, alkaloids, phenolic acids, and vitamins [22]. Plants of the genus *Baccharis* have been extensively studied for their therapeutic properties [23]. The most frequently identified compounds in the genus *Baccharis* are flavonoids, terpenoids, and quinic acid derivatives (chlorogenic acid derivatives) [24]. *Baccharis trimera* is popularly known as carqueja. This plant has leaves and stems are fused, and, consequently, therefore, they are termed by people as leaves. These leaves are used in folk medicine in developing countries and is used for various purposes such as treatment of liver problems [25], gastroprotection [26], hypoglycemia [27], and slimming and hypolipidemic action [28]. Flavonoids and chlorogenic acid have been identified as responsible for the anti-obesity [23,26,28,29,30,31] and antioxidant [27,31,32,33] action attributed to *B. trimera*.

*B. trimera* can promote weight reduction through the regulation of transcriptional factors and lipid and carbohydrate metabolism; decrease in cholesterol, low-density lipoprotein (LDL), triglyceride, and very-low-density lipoprotein (VLDL) levels; increased high-density lipoprotein (HDL) levels; decrease in glucose levels; and/or increased intestinal transit [23]. The antioxidant activity of *B. trimera* is related to its ability to suppress the formation of reactive species by inhibiting the enzymes involved in their production, and its ability to directly eliminate reactive species and to upregulate antioxidant defenses [34].

The nematode *C. elegans* is an experimental model widely used in biological and medical studies because of its numerous advantages over other animals. Among these characteristics, the following stand out: small size, simple and transparent body, high reproductive capacity, and low cost; these factors facilitate its easy cultivation in laboratories [35]. In addition to these factors, *C. elegans* has become, in recent years, a useful model for the study of fat storage [36,37], mainly due to the conservation of lipid metabolism pathways in mammals [38], as well as, to evaluate the intrinsic potential of extracts and biomolecules as potential antioxidant agents [33,39,40], since they have high homology of their genome with the mammalian genome, in relation to the phase I detoxification system (cytochrome p450), phase II [41], and phase III [42], which are important for studies of antioxidant activity.

Several genes and pathways have been identified to modulate stress resistance [39,43] and lipid metabolism [44,45] in *C. elegans*. Among the pathways involved in stress resistance are the FOXO/DAF-16 homolog and the Nrf-1/2 SKN-1 homolog. DAF-16/FOXO target genes include genes related to stress response/longevity. SKN-1 mediates the expression of genes involved in a wide range of detoxification processes. Regarding lipid metabolism, *C. elegans* has gene homologs that encompass a wide range of components of the regulatory cascade of lipid metabolism in mammals [46]. For example, in *C. elegans*, the TUB-1 ortholog is involved in lipid storage in these animals [47]. In addition, the factor NHR-49, homologous to the mammalian hepatocyte nuclear factor 4 alpha (HNF4α), regulates the expression of several genes involved in the β-oxidation and desaturation pathways of fatty acids [48,49].

Data on the mechanism of action of *B. trimera* extracts are still scarce and require further studies, including in vivo studies, such as those on the *Caenorhabditis elegans* model. Therefore, in the present study, the antioxidant effect, and the inhibitory capacity of the lipid accumulation of the aqueous extract of the leaves of *B. trimera* were investigated, using an animal model, *C. elegans*. In addition, we evaluated metabolic pathways that could be involved in the observed biological actions.

## 2. Materials and Methods

### 2.1. Materials

Gallic acid was purchased from CAQ Química Ind. E Com. (Diadma, SP, Brazil). Phenol was obtained from Reagen Quimibrás Indústrias Químicas S.A. (Rio de Janeiro, RJ, Brazil). Iron chloride, iron II sulfate, trichloroacetic acid, sulfuric acid, and Folin-Ciocalteau reagent were purchased from Merk (Darmstadt, Germany). Copper II sulfate was obtained from CRB–Cromato produtos químico Ltd.a (São Paulo, SP, Brazil). Monobasic sodium phosphate and dibasic sodium phosphate were purchased from CRQ (Diadema, SP, Brazil). 2,2-diphenyl-1-picrylhydrazyl (DPPH), 2′,7’-dichlorodihydrofluorescein diacetate (DCFH_2_-DA), trichloroacetic acid (TCA), Ethylenediaminetetraacetic acid (EDTA), 3-(4,5-Dimethylthiazol-2-yl)-2,5-Diphenyltetrazolium Bromide (MTT), nitroblue tetrazolium (NBT), methionine, pyrocatechol violet, riboflavin, ammonium molybdate, fluorodeoxyuridine (FUdR), copper sulfate, tert-butyl hydroperoxide (t-BOOH), sodium chloride, ascorbic acid, bovine serum albumin (BSA), D-glucose, Oil Red O, magnesium sulfate, and ferrozine were purchased from Sigma-Aldrich Co. (St. Louis, MO, USA). Penicillin and streptomycin were obtained from Fort Worth, TX, USA. Nematode Growth Medium (NGM): bacteriological agar and peptone (Kasvi, São José do Pinhais, PR, Brazil), cholesterol (Vetec, Jaragua do Sul, SC, Brazil), and calcium chloride (Synth, Diadema, SP, Brazil). Triglycerides were determined using a kit (LABTEST, Diagnostica S.A., Minas Gerais, Brazil).

The *C. elegans* strains N2 (wild-type strain) and EU1 (skn-1(zu67) IV/nT1 (IV;V) were obtained from the *Caenorhabditis Genetics Center* (CGC), University of Minnesota, USA (http://www.cbs.umn.edu/cgc) (accessed on 20 August 2022).

### 2.2. Acquisition of B. trimera Leaf Extract (BT)

*B. trimera* (Asteraceae) plants were obtained from Belo Horizonte (Minas Gerais, Brazil). This plant has fused leaves and stems and, consequently, they could not differentiate and easily separate from each other. People call these parts leaves and use them as medicine. Moreover, here, these parts were also called leaves and used to obtain the extract of *B. trimera*. Therefore, the selected leaves were dehydrated in an aerated oven at 45 °C for three days and then crushed. From the selected and crushed leaves, the extract was obtained by decoction, according to the protocol already described [31]. The sample (50 g) was boiled in 300 mL of water for 10 min, followed by filtration, freezing, and lyophilization (−45 °C; 0.12 mBar) (Freezone 4,5, Labconco Corporation Kansas City, MO, USA). The extract obtained from this process is hereby referred to as the BT.

### 2.3. Physicochemical Characterization of BT

Total phenolic compounds were quantitatively evaluated by the Folin–Ciocalteu colorimetric method using gallic acid as a standard. Total sugar content was determined by the phenol-sulfuric acid method [50], using D-glucose as a standard. The protein content was determined using Comassie blue R reagent [51], with albumin as a standard.

The analyses by high-performance liquid chromatography coupled to an ultraviolet spectrum scanning photodiode array detector (HPLC-UV-DAD) were carried out on an Agilent 1260 Chromatograph equipped with a 60 mm flow cell with a detection speed of 80 Hz and a detector by an array of ultraviolet and visible spectrum scanning photodiodes (range between 200 and 600 nm). For analysis on an analytical scale, a reversed-phase C18 Zorbax Eclipse plus column (150 × 4.6 mm) with a 3.5 μm particle diameter were maintained at 45 °C, with a mobile phase flow of 1.0 mL/min and 3 μL injection volume of samples, prepared at a concentration of 5 mg/mL. The wavelengths used to record the chromatograms were 254, 280, 325, and 352 nm. The gradient elution system consisted of water acidified with 0.1% acetic acid (eluent A) and acetonitrile (eluent B) in the following chromatographic run: 0 to 6 min, 10% B; 6 to 7 min, 10 to 15% B; 7 to 22 min, 15% B; 22 to 23 min, 15 to 20% B; 23 to 33 min, 20% B; 33 to 34 min, 20 to 25% B; 34 to 44 min, 25% B; 44 to 54 min, 25 to 50% B; and 54 to 60 min, 50 to 100% B.

### 2.4. Assessment of BT Cytotoxicity

The MTT assay measures the redox environment of the intracellular medium and is widely used to assess the toxicity of various compounds. Assays were performed as previously described [52]. To this end, murine fibroblasts (3T3) were cultured in culture flasks in Dulbecco’s modified Eagle medium (DMEM) with 10% (*v*/*v*) fetal bovine serum, 100 μg/mL streptomycin, and 100 IU/mL penicillin until the experiments were performed. The cells were transferred to sterile 96-well plates at a density of approximately 5 × 10^3^ cells/well and incubated for 24 h at 37 °C and 5% CO_2_. After this time, the medium was changed to serum-free DMEM for synchronization of all the cells at G0 (active, but not dividing cells). The cells remained in this condition for 24 h. Subsequently, the medium was removed, and medium containing fetal serum (10%) and BT at different concentrations (from 0.1 to 1 mg/mL) was added. After 24 h of incubation, cellular traces were removed by washing cells with phosphate-buffered saline (PBS). A serum-free culture medium containing 12 mM MTT was added to determine the ability of the cells to reduce MTT. The cells were then incubated for 4 h at 37 °C and 5% CO_2_. After this period, the medium was removed and to solubilize the reduced MTT product, 100 μL of ethanol was added to each well and mixed thoroughly using a multichannel pipettor. After 15 min of ethanol addition, the absorbance at 570 nm was measured using a microplate reader (Thermo LabSystems, Franklin, MA, USA).

### 2.5. In Vitro Antioxidant Assays

The assays were performed as previously described [53] to analyze the in vitro antioxidant activity of BT, as well as the total antioxidant capacity (TAC), reducing power, iron chelation, copper chelation, superoxide radical scavenging and hydroxyl radical scavenging. The absorbance of the solution was measured using an Agilent Bio Teck Epoch microplate spectrophotometer (Agilent Technologies, Santa Clara, CA, USA).

Tubes containing BT (from 0.1 to 2.0 mg/mL) and reagent solution (0.6 M sulfuric acid, 28 mM sodium phosphate, and 4 mM ammonium molybdate) were incubated at 95 °C for 90 min. After the mixture was cooled to room temperature, the absorbance of each solution was measured at 695 nm against a blank. After performing the experiment, the concentration at which the absorbance values no longer increased was identified. In the case of BT, from 1.0 mg/mL, no further alterations in the absorbance values were identified. Therefore, this was the concentration chosen to calculate the total antioxidant capacity (TAC), as described earlier [53]. Ascorbic acid was used as standard. The results were expressed as acid ascorbic equivalent per gram of BT.

The reducing power was evaluated after adding 4 mL of the solution containing the BT (0.1, 0.5, 1.0 and 2.0 mg/mL) to phosphate buffer (0.2 M; pH 6.6) and potassium ferricyanide (1%). The mixture was incubated for 20 min at 50 °C. The reaction was stopped by the addition of 10% trichloroacetic acid, distilled water, and iron chloride. The absorbance of the solution was measured at a wavelength of 700 nm, and ascorbic acid was used as a positive control.

The iron chelating ability of BT was evaluated as described earlier [53]. Briefly, BT (0.1, 0.5, 1.0 and 2.0 mg/mL) was added to the reaction mixture containing FeCl_2_ (0.05 mL, 2 mM) and ferrozine (0.2 mL, 5 mM). The mixture was shaken and incubated for 10 min. at room temperature, and the absorbance of the mixture was measured (562 nm) against a blank (ultrapure water). The control was the reaction mixture without polysaccharides. EDTA (ethylenediaminetetraacetic acid) was used as positive control.

The chelating effect was calculated using the corresponding absorbance (A) in the formula given below, where the control is the absorbance in the absence of chelating agents:

Chelating Effect (%) = (Acontrol − Asample/Acontrol) × 100(1)

The copper chelation test was performed in 96-well microplates with a reaction mixture containing different concentrations of BT (0.1, 0.5, 1.0 and 2.0 mg/mL), pyrocatechol violet (4 mM), and copper II sulfate pentahydrate (50 mg/mL). The solution in each well was mixed using a micropipette, and the absorbance was measured at 632 nm. The chelating effect was calculated using the corresponding absorbance in the formula given below, where the blank represents the absorbance in the absence of chelating agents. EDTA (ethylenediaminetetraacetic acid) was used as positive control.

Chelating Effect (%) = (Acontrol − Asample/^A^control) × 100 (2)

For the hydroxyl radical scavenging assay, a solution (10 mM FeSO_4_·7H_2_O, 10 mM EDTA, 2 mM sodium salicylate, 30% H_2_O_2_) was used to generate these radicals. Phosphate buffer (150 mM, pH 7.4) was used as a negative control and gallic acid as a positive control. BT (0.5, 1.0, 1.5 and 2.0 mg/mL) was incubated with the solution (1 h, 37 °C). Subsequently, hydroxyl radical scavenging was detected using a microplate reader (510 nm).

Superoxide Radical Scavenging Activity Assay is based on the capacity of BT to inhibit the photochemical reduction of NBT in the riboflavin–light–NBT system. Each 3 mL of reaction mixture contained 50 mM phosphate buffer (pH 7.8), 13 mM methionine, 2 mM riboflavin, 100 mM EDTA, NBT (75 mM) and 1 mL BT solution (0.1, 0.5, 1.0 and 2.0 mg/mL). After the production of blue formazan, the increase in absorbance at 560 nm after 10 min illumination from a fluorescent lamp was determined. The entire reaction assembly was enclosed in a box lined with aluminum foil. Identical tubes with the reaction mixture were kept in the dark and served as blanks. Gallic acid was used as positive control.

The antioxidant activity was also determined by the ability of the antioxidants present in the BT to scavenging the radical DPPH [54]. BT was tested in different concentrations (0.1, 0.5, 1.0, and 2.0 mg/mL).

### 2.6. Assays with C. elegans

#### 2.6.1. Conditions for the Maintenance and Treatment of *C. elegans*

Wild-type *C. elegans* (N2) and mutant skn-1(zu67) strains were used in this study. The worms were routinely propagated at 20 °C on plates containing Nematode Growth Medium (NGM) seeded with *Escherichia coli* OP50 at 20 °C according to Brenner [55].

The synchronization of N2 animals was done using the alkaline lysis protocol. This protocol is based on treating pregnant adult hermaphroditic worms with lysis solution (1.0 mL of NaOH solution (10.0 M), 4.0 mL of sodium hypochlorite (2%), and 5.0 mL of deionized water) for a maximum of 10 min. Embryos resistant to lysis solution treatment were collected and placed in liquid M9 solution (3.0 g KH_2_PO_4_, 6.0 g Na_2_HPO_4_, 5.0 g NaCl, 1.0 mL MgSO_4_ (1.0 M), and 800 mL distilled water) for 12 h in the absence of food. The worms (synchronized in the L1 stage) were transferred to NGM plates containing or not containing BT (1.0 or 5.0 mg/mL).

To figure out the role of some genes in antioxidant and anti-obesity effect of BT, these genes were inhibited using the technique of RNA interference (RNAi) by feeding as described earlier [33]. RNAi can be induced by feeding worms bacteria expressing dsRNA. This which consists of cloning cDNA corresponding to the gene into plasmid pL4440 that is then transformed into the *E. coli* HT115 bacteria, providing IPTG inducible expression of the phage T7 RNA polymerase. The HT115 strain lacks the Rnc gene, which encodes RNase III and is therefore deficient in degrading dsRNA. The RNAi food is prepared and seeded onto NGM plates containing IPTG. Then, the animals are placed onto the RNAi food, as described billow.

Clones containing RNAi were cultured with 12.5 µg/mL tetracycline and 100 µg/mL ampicillin. Subsequently, the cultures were diluted in LB medium supplemented with 60 μg/mL of ampicillin and cultivated at 37 °C under shaking overnight. The following day, a 1:10 dilution of each culture was performed and grown until an optical density (OD) between 0.6–0.8 was obtained. Subsequently, the inoculums were centrifuged (5000 rpm, 8 min, 25 °C), the supernatant was aspirated, and the bacterial pellet was suspended with LB (1 mL) plus ampicillin (1 µL) and IPTG (1 µL). These inoculums were seeded on plates with NGM and BT (5 mg/mL) and kept for 48 h at 20 °C to dry and form a layer of bacteria that will serve as food for the animals. Synchronized L1 larvae were then placed in these plates that contained a layer of HT115 with plasmid pL4440 containing the exonic sequence of skn-1, daf-16, *tub-1*, *nhr-49*, or with HT115 with plasmid pL4440 only (control), until they reached the L4 stage.

#### 2.6.2. Bacterial Growth Assay

To monitor the proliferation of *E. coli* OP50, OD readings were performed in a spectrophotometer (Biochrom Libra S22, Cambridge, UK). *E. coli* OP50 bacteria were suspended with LB medium contained BT (1.0 and 5.0 mg/mL). Optical density (OD) equal to 0.05 was considered the zero point. The tubes were incubated in a shaker (MA 420) at 37 °C and the OD was measured every 60 min for 4 h. Results were expressed as relative levels. The OD of *E. coli* OP50 was compared at each point with the OD at the zero point of each condition.

#### 2.6.3. Evaluation of BT Toxicity in *C. elegans*

The synchronized worms at the L1 stage and cultured on NGM medium plates were separated into groups and subjected to treatment with BT (1.0 and 5.0 mg/mL) for 48 h at 20 °C. In the control group, nematodes were cultivated in NGM containing *E. coli* OP50. In total, 30 worms from each group were photographed, and body length was measured using ImageJ—Image Processing and Analysis in Java software (ImageJ 1.51j8, National Institute of Mental Health, Bethesda, MD, USA, https://imagej.nih.gov/ij/ accessed on 20 August 2022). To quantify the hatching rate, wild-type (N2) *C. elegans* eggs obtained by alkaline synchronization were used. These were placed in Petri dishes with NGM and OP50 with or without treatment. The plates were then placed in an incubator at 20 °C for 29 to 38 h. After this period, the plates were placed at 4 °C, protected from light for 24 h, which induced reduced mobility or death of the animals and facilitated the counting of animals in a stereoscopic magnifying glass. Thirty animals per plate were used. Three independent experiments were carried out, and in each one, triplicates were used.

#### 2.6.4. Intracellular Accumulation of ROS in *C. elegans*

Intracellular ROS production in *C. elegans* was measured using a 2 mM 2′,7′-dichlorodihydro-fluorescein diacetate (H2DCF-DA) fluorescent probe. Wild-type N2 animals were synchronized in L1 and they were treated or not with BT (1.0 and 5.0 mg/mL) for 48 h at 20 °C. Then, approximately 40 animals from each group, in biological triplicate, were transferred to a 96-well plate containing H2DCF-DA. Fluorescence quantification was performed in a microplate reader (GloMax^®^-Multi Detection System, Promega, Madison, WI, USA), with excitation at 490 nm and emission at 510–570 nm. Five readings were performed, with an interval of 30 min between each reading. Three independent experiments were carried out, and in each one, triplicates were used.

#### 2.6.5. Oxidative Stress Survival Assay

The worms (L1) grew in culture medium that contained BT (1.0 or 5.0 mg/mL) or did not (control group). After 48 h, 50 worms (now in L4) from each group were transferred to a new plate containing medium and t-BOOH (7.7 mM, final concentration) for the induction of oxidative stress. The worms were evaluated and classified as dead when they were not moving, even after repeated touches with a platinum handle. Viability was assessed after 3, 15, 21, 24, 27, 30, 33, 36, 39 and 42 h. In all experiments, the worms were cultured on NGM plates containing FUdR, to prevent progeny growth. Three independent experiments were carried out, and in each one, triplicates were used.

#### 2.6.6. Oil Red O Staining

The lipid content in *C. elegans* was quantified by Oil Red O staining according to a previously described protocol [56]. The worms were washed three times with M9 buffer (0.3% KH_2_PO_4_, 0.6% Na_2_HPO_4_, 0.5% NaCl, and 1 mM MgSO_4_) and fixed with 40% isopropanol for 3 min. The worms were centrifuged (560 rpm, 30 s), washed, and stained with Oil Red O (Sigma-Aldrich, St. Louis, MO, USA) for 2 h under gentle agitation (30 rpm), and slides were prepared. At least 20 nematodes per group in each experiment (total n per group = 60 animals) were photographed under an optical microscope at 10× magnification (using AMScope 3.7 software) and analyzed using ImageJ software as pixels in the red component of RGB normalized to body size. The results are expressed as the variation in the intensity relative to the control. Subsequently, similar tests were performed with wild-type N2 animals but treated with BT (5.0 mg/mL) in the presence or absence of high glucose, using the RNAi technique for *tub-1* and *nhr-49* genes.

#### 2.6.7. Triglyceride Quantification Assay

To quantify triglycerides, 2000 animals were seeded and treated for 48 h with samples containing BT (1.0 and 5.0 mg/mL) in the presence or absence of high glucose. After treatment, nematodes were collected and washed three times with M9 buffer to remove all bacteria and lysed in 0.05% Tween 20 in a sonicator (Sonics VCX-130 Vibra-Cell Ultrasonic Liquid, UniGreenScheme, Caerphilly, UK) for 3 min. Triglyceride content was determined using a commercial kit (LABTEST, Diagnostica S.A., Lagoa Santa, Minas Gerais, Brazil) according to the manufacturer’s protocol. To normalize the triglyceride content, the total protein content of the sonicated animals was quantified using NanoVue Plus equipment (Biochrom, Holliston, MA, USA), following the manufacturer’s specifications, and the results were expressed relative to the negative control.

### 2.7. Statistical Analyzes

Three independent trials were performed. Statistical analyses were performed using GraphPad Prism (v.6.01) (San Diego, CA, USA). Results were represented as the mean ± standard error of mean (SEM) of three individual experiments. Analysis of variance (ANOVA) followed by Tukey’s post-hoc test was used to compare the means between the groups analyzed. Survival curves were made using the log-rank test (Mantel-Cox). For all tests, statistical significance was set at *p* < 0.05.

## 3. Results

### 3.1. Characterization of BT

**Figure 1:**
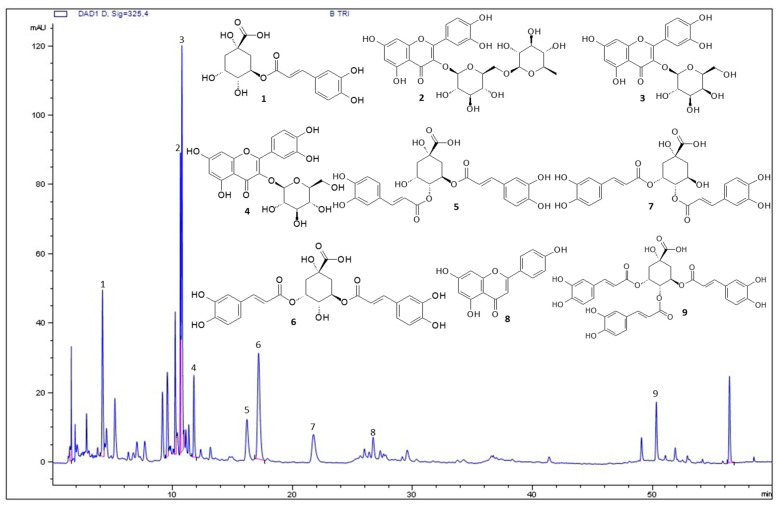
Chromatogram of *Baccharis trimera* leaf extract (BT) obtained from HPLC-UV-DAD. (1) 5-caffeoylquinic acid; (2) rutin; (3) hyperoside; (4) isoquercetin; (5) 4,5-dicaffeoylquinic acid; (6) 3,5-dicaffeoylquinic acid; (7) 3,4-dicaffeoylquinic acid; (8) apigenin; (9) 3,4,5-tricaffeoylquinic acid. The other peaks of the chromatogram were not identified, however, these compounds pertain to flavonoid and chlorogenic acid derivatives classes. BT: *Baccharis trimera* aerial part extract; HPLC-UV-DAD: high-performance liquid chromatography coupled to an ultraviolet spectrum scanning photodiode array detector.

The BT chromatographic profile (Figure 1) was obtained using HPLC-UV-DAD. The compounds were identified based on the retention times of commercial standards or compounds previously isolated and described by nuclear magnetic resonance (NMR) analysis [42]. Nine compounds were identified in the BT extract: rutin, hyperoside, isoquercetin, and apigenin (peaks 2, 3, 4 and 8, respectively), and chlorogenic acid derivatives including 5-caffeoylquinic acid, 4,5-dicaffeoylquinic acid, 3,5-dicaffeoylquinic acid, 3,4-dicaffeoylquinic acid, and 3,4,5-tricaffeoylquinic acid (peaks 1, 5, 6, 7, and 9, respectively).

### 3.2. Assessment of BT Toxicity

**Figure 2:**
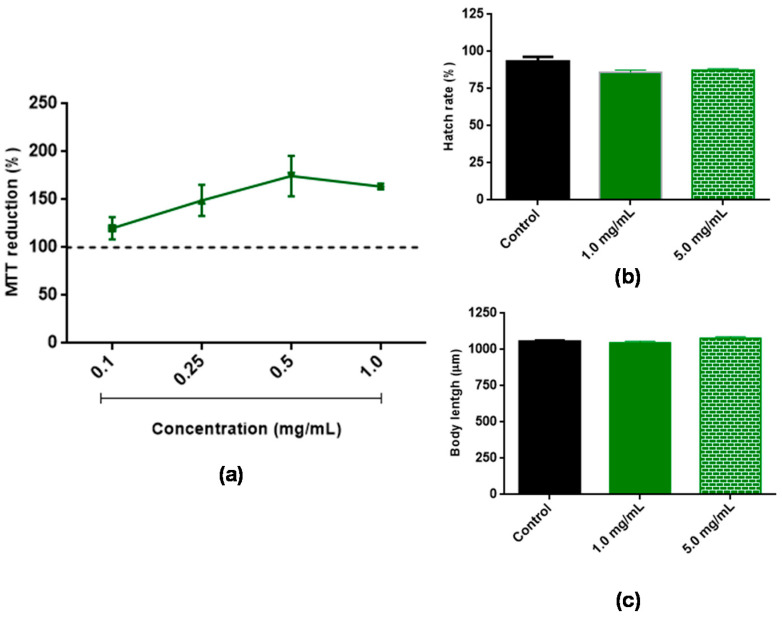
Evaluation of the toxicity of *Baccharis trimera* leaf extract (BT). (**a**) Effect of BT on MTT reduction capacity of 3T3 cells. (**b**) Effect on hatch rate of *Caenorhabditis elegans* eggs. (**c**) Effect on body length of worms. Cells were cultured in the presence of different concentrations (0.1–1.0 mg/mL) of BT for 24 h. The animals were exposed to 1.0 and 5.0 mg/mL of BT. Cell viability was measured using the MTT test. The dotted line represents the negative control. The letter a indicates significant differences between the different concentrations and the control according to ANOVA followed by the Tukey test (*p* < 0.01). MTT: 3-[bromide 4,5-Dimethyl-thiazol-2-yl]-2,5-diphenyltetrazolium; ANOVA: analysis of variance.

To investigate the cytotoxicity of BT, 3T3 cells were treated with different concentrations of BT, and their ability to reduce MTT was evaluated as described in the Methods section. BT did not show cytotoxicity at any of the concentrations evaluated (Figure 2a). The possible toxicity of BT against *C. elegans* was also evaluated. After 48 h of exposure to 1.0 and 5.0 mg/mL BT, no significant changes were observed in the evaluated parameters including hatching rate (Figure 2b) and body length (Figure 2c). These results indicate that BT did not exhibit toxicity under the conditions evaluated.

### 3.3. Antioxidant Assays In Vitro

To evaluate the in vitro antioxidant activity of BT, seven antioxidant assays were performed: TAC, reducing power, metal chelation (iron and copper), superoxide radical scavenging, hydroxyl radical scavenging, and DPPH.

**Figure 3:**
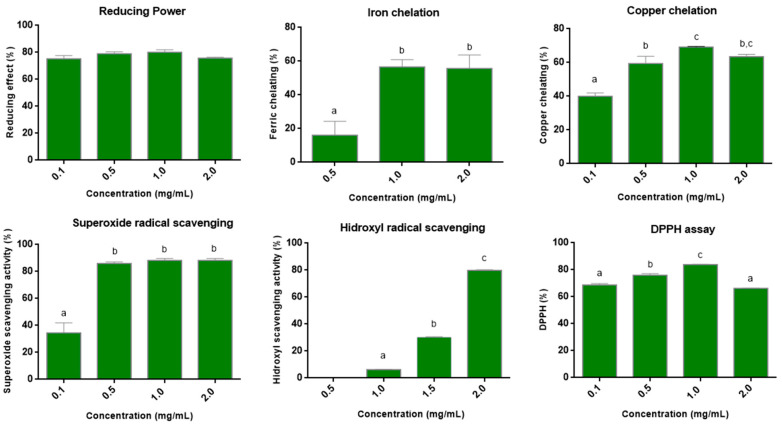
Antioxidant activity of *B. trimera* leaf extract (BT). Reducing power; Iron chelation; Copper chelation; Superoxide radical scavenging; scavenging of hydroxyl radicals and DPPH Assay. Assays were performed in triplicate, and data were analyzed using ANOVA and Tukey’s test (*p* < 0.05). Different letters (a, b, c) correspond to significant differences in the statistical analysis.

The TAC test showed activity of 57.8 equivalent grams of ascorbic acid/g of BT. In the test of reducing power, at the lowest concentration evaluated (0.1 mg/mL), an activity ~80% was already observed (Figure 3). Furthermore, this activity did not change with increasing BT concentration (0.5 to 2.0 mg/mL). In the DPPH radical scavenging tests, a value of 60% was observed with 0.1 mg/mL of BT, and the maximum value (~80%) was observed with 1.0 mg/mL. This value did not change with the increase in the concentration of BT.

Regarding the chelating capacity of metals (Figure 3), BT showed the ability to chelate both copper and iron. Furthermore, in both cases, the maximum activity was observed at 1.0 mg/mL. Chelation of 56.6% and 69.2% were observed for iron and copper, respectively.

In the superoxide radical scavenging assay, a dose-dependent effect was observed, which stabilized (~86%) around 0.5 mg/mL (Figure 3). In the hydroxyl radical scavenging test, in the lowest concentrations evaluated, a low activity was observed. However, with 2.0 mg/mL, an activity around 80% was obtained.

### 3.4. Bacterial Growth Assay

Before the in vivo antioxidant tests, the effect of BT on the growth of the bacterium *E. coli* OP50, which is used as a food source for C. elegans, was evaluated. BT at concentrations of 1.0 and 5.0 mg/mL did not show antibacterial activity against *E. coli* OP50.

### 3.5. Antioxidant Assays In Vivo Using C. elegans

The worm *C. elegans* was used to evaluate the antioxidant potential of BT in vivo. For this, concentrations of 1.0 mg/mL (based on in vitro tests) and 5.0 mg/mL were chosen. The latter was necessary due to the thick cuticle present in the worms, which makes it difficult to absorb substances.

**Figure 4:**
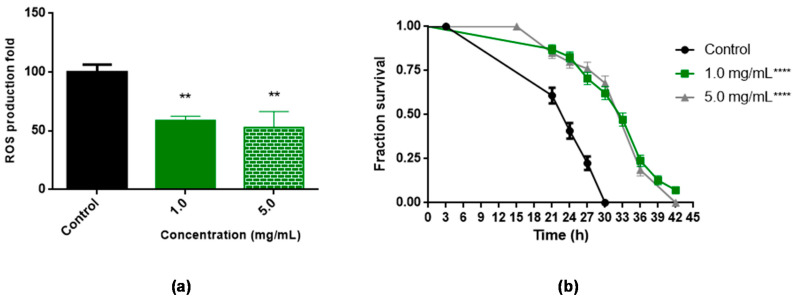
Effect of *B. trimera* (BT) leaf extract on the *C. elegans* model. (**a**) Levels of reactive oxygen species (ROS) in wild-type (N2) strains treated with BT for 48 h. Three independent experiments were performed, with three replicates per treatment (n = 40). (**b**) Survival of wild-type (N2) animals under oxidative stress conditions (approximately 120 animals/group). The worms were treated with BT from stage L1 to stage L4. The BT treatment was compared to the control using the Log-rank test **** *p* < 0.0001 and ** *p* < 0.001 with data evaluated by ANOVA and Tukey’s test.

Initially, it was evaluated whether BT was able to reduce intracellular levels of ROS. The results demonstrated that BT decreased ROS levels by 58.8% and 52.9%, at concentrations of 1.0 mg/mL and 5.0 mg/mL, respectively, compared to the control (Figure 4a).

Subsequently, it was evaluated whether treatment with BT increased the survival of *C. elegans* under conditions of oxidative stress induced by t-BOOH. As seen in Figure 4b, treatments with 1.0 and 5.0 mg/mL BT increased *C. elegans* survival under stress conditions (Figure 4b). The BT promoted a significant shift to the right in the survival curve under induced acute oxidative stress at the concentrations evaluated when compared to the control, increasing worm survival from 30 to 42 h (*p* < 0.0001) (Figure 4b). The increase in average lifespan when compared to the control group (*p* < 0.0001) can be seen in Table 1.

**Figure 5:**
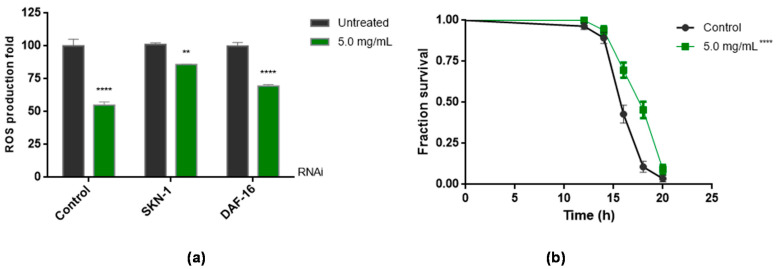
Effect of *B. trimera* leaf extract (BT) on intracellular ROS levels and oxidative stress resistance in *C. elegans*. (**a**) Contribution of SKN-1 and DAF-16 to ROS accumulation. RNAi animals were treated with samples at a concentration of 5 mg/mL for approximately 48 h from L1. ROS production was measured using the DCFH_2_-DA dye. Results are expressed as mean DCFH_2_-DA fluorescence levels ± SEM values. **** *p* < 0.0001 and ** *p* < 0.001, compared to the respective untreated RNAi by ANOVA, followed by Tukey’s test. (**b**) Survival curves of skn-1 (zu67) mutant animals under oxidative stress conditions. The animals were treated with 5.0 mg/mL of BT. Subsequently, they were subjected to 7.7 mM of t-BOOH. Survival was checked every 2 h **** *p* < 0.0001 in relation to the respective control by the Log-rank test (Mantel-Cox). Control—worms fed with the empty plasmid.

To elucidate whether the ROS reduction was related to direct or indirect mechanisms of action, ROS quantification was performed in wild animals submitted to the RNAi technique for the transcription factors SKN-1 and DAF-16. The results are shown in Figure 5.

In both cases, there was a decrease in the amount of ROS in the worms when they were exposed to BT (Figure 5a). However, the effect of BT was smaller in SKN-1 animals than in DAF-16 animals.

Figure 5b shows that the skn-1 gene mutant animals had a significantly higher survival rate (*p* < 0.0001) than the control group animals.

### 3.6. In Vivo Assays to Assess Lipid Accumulation Using C. elegans

**Figure 6:**
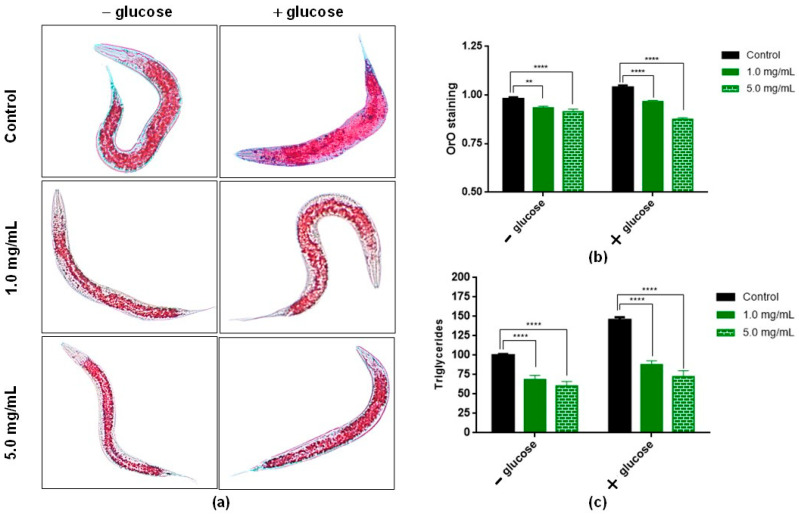
Effect of *Baccharis trimera* leaf extract (BT) on fat accumulation in wild-type *C. elegans* (N2) by Oil Red O staining and triglyceride quantification. L1 stage worms were treated with BT (1.0 or 5.0 mg/mL) for 48 h in absence or with glucose co-treatment. (**a**) Images were obtained from worms stained with Oil Red O. (**b**) Fat accumulation measured in red pixels normalized by body size. At least 20 animals per group were analyzed in each experiment in triplicate (total n = 60 animals). (**c**) Quantification of triglycerides. Data represent the mean ± standard deviation of three independent experiments. ** *p* < 0.01 and **** *p* < 0.0001, being statistically significant in relation to the control, according to ANOVA followed by a Tukey test. BT: *Baccharis trimera* aerial part extract; ANOVA: analysis of variance.

To assess the role of BT in lipid accumulation, wild-type (N2) worms were treated with BT in the presence or absence of a high amount of glucose. The results of Oil Red O staining and triglyceride quantification showed that after 48 h of treatment with BT (1.0 and 5.0 mg/mL), the accumulation of fat in *C. elegans* decreased in the absence or presence of high amounts of glucose. Figure 6a shows images of animals stained with Oil Red O. In the presence or absence of glucose, the animals treated with BT (1.0 and 5.0 mg/mL) showed less intense staining compared to the respective control. This result was confirmed by the analysis presented in Figure 6b. Here, the results obtained showed that after 48 h of treatment with 1.0 and 5.0 mg/mL, fat accumulation was significantly reduced in all groups when compared to the control.

To assess whether BT plays a role in lipid accumulation, triglyceride levels were quantified in *C. elegans* in the presence or absence of a high amount of glucose. The results after 48 h of treatment with BT (1.0 and 5.0 mg/mL) are shown in Figure 6c. It was observed that BT reduced triglyceride content in worms. In the low-glucose group, there was a reduction of 31.5% and 39.7% with the use of BT at 1.0 and 5.0 mg/mL, respectively. In glucose-enriched diets, the reduction in triglyceride levels was 12.2% and 27.6% with the use of BT at 1.0 and 5.0 mg/mL, respectively.

**Figure 7:**
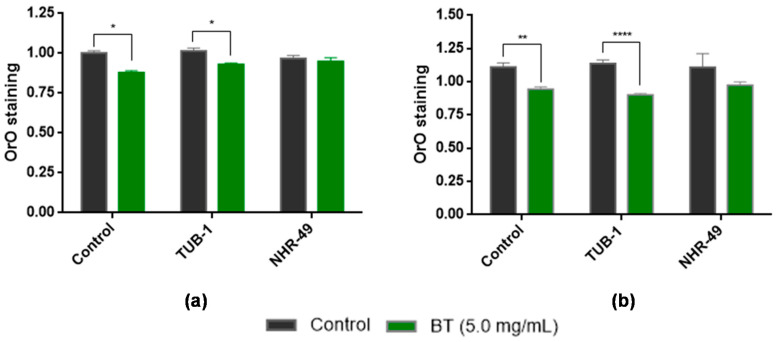
Contribution of TUB-1 and NHR-49 transcription factors to lipid accumulation in animals treated with *Baccharis trimera* leaf extract (BT) at 5.0 mg/mL. Wild-type animals (N2) in L1 stage were treated with BT for 48 h with low glucose (**a**) and high glucose (**b**). **** *p* < 0.0001, ** *p* < 0.01 and * *p* < 0.05, being statistically significant in relation to the control, according to ANOVA, followed by the Tukey test. BT: *Baccharis trimera* aerial part extract; ANOVA: analysis of variance. Control—worms fed with the empty plasmid.

To elucidate the mechanism of action of BT, which is involved in reducing the accumulation of lipids, tests were carried out in animals subjected to RNAi for TUB-1 and NHR-49 transcription factors. The results showed that the *tub-1* knockdown animals showed a significant reduction in the accumulation of lipids when compared to the control group (Figure 7); that is, the effect of BT was not abolished in these animals. In relation to *nhr-49* knockdown animals, no statistical difference was observed between the treated groups and the control. This result indicates that the reduction in lipid accumulation may be related to the transcription factor, NHR-49.

## 4. Discussion

The biological activity of *B. trimera* is attributed to its phytochemicals. HPLC-UV-DAD analysis showed that BT contains different types of phenolic compounds (Figure 1). This result is consistent with those of previous studies. Phytochemical analyses of different extracts of *B. trimera* revealed the presence of rutin [57,58,59], apigenin [60,61,62], and isoquercetin [57,60,61,63]. Another class of natural products identified by HPLC-UV-DAD is caffeoylquinic acid. This finding corroborates the results of a previous study [31], that highlighted the presence of 5-caffeoylquinic acid (chlorogenic acid) in the extract of *B. trimera*. In summary, the results showed that the phytochemicals found here have already been described in *B. trimera* indicating that they can be used as markers of the quality of extracts obtained in the future.

Several of the BT phytochemicals identified here are pointed out as antioxidant agents. The antioxidant activity of extracts of *B. trimera* has already been demonstrated by different authors [33,64,65]. In fact, our group demonstrated the antioxidant activity of BT in a previous study [31]. However, as here, BT comes from a new extraction, its in vitro antioxidant activity was again evaluated. Furthermore, as antioxidants can act at any stage of the oxidative process (initiation, propagation and termination), their quality is also evaluated by the number of steps taken during which these compounds can exert their activity [66,67]. The antioxidant activity of BT was evaluated in vitro using seven different tests, which together evaluate the activity of an antioxidant agent in the different stages of the oxidative process.

Regarding the TAC and reducing power tests, the results obtained here were similar to those shown in a previous study [31]. These tests showed that BT was able to donate electrons and/or hydrogen atom in different chemical microenvironments. BT was able to scavenge superoxide radicals, as well as being able to chelate metals (iron and copper). These properties are desirable in an antioxidant agent, as Cu^+2^ and Fe^+2^ ions can react with H_2_O_2_ and generate hydroxyl radicals through Fenton and Haber-Weiss reactions. Both the superoxide radical and the hydroxyl radical can cause damage to proteins, lipids, and DNA, which consequently causes cellular and tissue damage [68]. Furthermore, BT was able to scavenge the hydroxyl radical directly. The elimination of this radical is highly desirable since its presence is associated with several diseases [69]. Therefore, the data obtained here show that BT, through direct or indirect mechanisms, can combat two very important ROS for the formation of oxidative stress. Furthermore, BT showed activity above 80% in the DPPH assay. These data showed that BT has the potential to act as an antioxidant compound at different stages of the oxidative process. Indeed, the antioxidant potential of BT comes from the different phytochemicals present in its constitution, since they have different mechanisms of antioxidant action, and, therefore, they may be acting together to give BT the antioxidant activity observed in the different tests.

Considering that BT showed excellent antioxidant activity in vitro, the nematode *C. elegans* was used as a model to evaluate the in vivo antioxidant potential of BT. First, it was evaluated whether BT would interfere with the proliferation of *E. coli* OP50 and it was found that BT did not interfere with the proliferation of these bacteria.

*E. coli* OP50 is food for the worms and changing the amount as well as the viability of the bacteria changes the lifespan of *C. elegans* [70]. Therefore, if BT affected the proliferation of this bacterium, it would affect the data observed in the following trials. As BT did not interfere with the proliferation of *E. coli*, OP50 was followed up with the experiments with *C. elegans*.

BT reduced intracellular ROS levels in the worms (Figure 4a) and these results corroborate the data obtained immediately (Figure 3). The reduction of intracellular levels of ROS are often associated with the survival rate under stress conditions of these animals [71]. This fact was confirmed with data presented in Figure 4b. Although we have not identified other papers that have evaluated the antioxidant effect of aqueous extracts of *B. trimera* on *C. elegans*, a paper that evaluated a hydroalcoholic extract of *B. trimera* in *C. elegans* also demonstrated that this extract reduced ROS levels in *C. elegans* and increased the survival of these worms [33]. A new blackberry cultivar (BRS Xingu) decreased ROS levels in the nematodes by 30.6% after exposure. BRS Xingu containing several phenolic compounds includes 5 anthocyanins, 5 phenolic acids, and 5 non-anthocyanin flavonoids [72]. Despite this, BT was more effective than BRS Xingu in decreasing ROS levels in *C. elegans.* In another study [73], nanoencapsulated phenolic compounds from Shiraz grapes showed that nanoparticles acted as pro-oxidants with no resistance to oxidative stress and caused decreased longevity. These data demonstrate that the presence of polyphenols in the extracts is not a guarantee of protection against oxidative stress, and they also show the protective potential of BT against ROS.

Phytochemicals present in plants have the potential to directly modulate intracellular oxidative stress by eliminating reactive species. Furthermore, they are also able to modulate oxidative stress indirectly, in this case, for example, modulating signaling pathways related to oxidative stress defense mechanisms [74]. In *C. elegans*, these defense mechanisms are, at least in part, a result of the activation of transcription factors such as DAF-16 and SKN-1 [75]. Data have shown the potential for antioxidant compounds to activate both SKN-1 and DAF-16 in *C. elegans*, suggesting that these transcription factors mediate beneficial antioxidant-induced responses such as increased oxidative stress resistance and lifespan [43]. Thus, to begin to understand BT antioxidant mechanisms of action, we performed the ROS quantification in worms submitted to the RNAi technique for the transcription factors SKN-1 and DAF-16 (Figure 5a) and later, the assay of stress resistance in skn-1 (zu67) mutant animals (Figure 5b and Table 1).

The data showed that both the worms exposed to the iRNAs and the mutated worms continued to feel the antioxidant effects of BT, decreased ROS, and increased survival rate. This indicates that the antioxidant action of BT, that is, of its phytochemicals, is a direct action on the reactive species. A similar result was observed with a hydroethanolic extract of *B. trimera* [33]. In this paper, the authors demonstrated that the antioxidant action of the extract of *B. trimera* was independent of transcription factors.

Thus, the results found suggested that the transcription factors SKN-1 and DAF-16 are not involved in the antioxidant action of BT, supporting the idea of the direct action provided by the phytochemicals present in BT.

Oxidative stress plays a critical role in obesity and is related to the causes and mediating factors of this pathological state. ROS play a direct role in adipogenesis, and oxidative stress modulates several factors involved in obesity, including genetic factors, gut microbiota, insulin action, ghrelin, inflammation, adipokines, leptin, and the hypothalamic axis [76]. On the other hand, obesity causes systemic oxidative stress through different mechanisms, including oxidative phosphorylation, superoxide generation from NADPH oxidases, glyceraldehyde auto-oxidation, polyol, and hexosamine pathways, and protein kinase C activation [77]. Thus, in view of the close relationship between oxidative stress and obesity, the ability of BT to reduce lipid accumulation in *C. elegans* was also evaluated here.

The presence of BT decreased lipid accumulation in worms, even in the presence of high amounts of glucose. The values obtained here were similar than that observed in *C. elegans* exposed to resveratrol (phenolic compound), that had anti-obesity activity with a 31% reduction in mean fat content [78]. In addition, these data corroborate data related to the hypoglycemic activity of *B. trimera*. One study tested ethanolic and aqueous extracts from the aerial parts of *B. trimera*. Only the aqueous fraction of *B. trimera* at 2000 mg/kg reduced blood glucose in diabetic rats after a seven-day treatment, indicating the potential antidiabetic activity of *B. trimera* [79]. Data from a methanolic extract of *B. trimera* showed reduced weight gain and decreased serum cholesterol levels in male Wistar rats [28]. In relation to human experiments, the anti-obesity activity of *B. trimera* has been evaluated in a randomized clinical trial. The use of capsules containing 500 mg of dehydrated *B. trimera* significantly reduced the percentage of fat and fat weight in the patients compared to the control group [29].

To determine the mechanisms involved in the ability of BT to inhibit fat accumulation in *C. elegans*, key genes involved in lipid metabolism were analyzed using RNAi. *C. elegans* has gene homologs that span a wide range of components of the mammalian fat regulatory cascade, including hormone regulators, differentiation factors, and biosynthetic enzymes [46]. *C. elegans* TUB-1 is associated with vertebrate tubby-like proteins [80,81]. Deletion of *tub-1* in *C. elegans* leads to the increased storage of triglycerides, the main type of fat in worms. Studies have shown that TUB-1 interacts with the Rab GTPase (Rab GAP)-activating protein, RBG-3, to regulate fat storage [45]. RBG-3 promotes hydrolysis of RAB-7 GTPase, an important effector of endocytosis. Together, these pathways can regulate the degradation of molecules or receptors in neurons. Neuroendocrine signals generated in neurons regulate fat storage in the gut [45]. As shown in Figure 5, the *tub-1* knockdown animals showed a significant reduction (*p* < 0.05) in the accumulation of lipids when exposed to BT in the same manner as the animals in the control group, which led to the conclusion that TUB-1 is not involved in the mechanisms of lipid inhibition by BT.

In addition to TUB-1, *C. elegans* contains the transcription factor, NHR-49. The data presented in Figure 4 shows that the mechanism of action of BT as an anti-obesity agent is dependent on NHR-49. NHR-49, despite its sequence relationship with HNF4α, has biological activities similar to those of mammalian peroxisome proliferator-activated receptors (PPARs), implying an evolutionarily conserved role for NHRs in modulating consumption and fat composition [44].

PPARs are a subfamily of three ligand-inducible transcription factors that belong to the nuclear hormone receptor superfamily. By binding to PPAR-responsive regulatory elements (PPRE), these receptors heterodimerize with the retinoid X receptor (RXR) and control a group of genes involved in adipogenesis, lipid metabolism, inflammation, and maintenance of metabolic homeostasis [45]. In mammals, three different isoforms of PPARs have been described: PPAR-α, PPAR-β/δ, and PPAR-γ. PPAR-α is found in tissues that show high levels of β-oxidation (kidneys, liver, healing, and muscle). PPAR-β/δ is found in all tissues, and PPAR-γ is found at high levels in adipocytes [82].

## 5. Conclusions

The extract of the leaves of *B. trimera*, rich in phenolic compounds, mainly rutin, hyperoside, and 5-caffeoylquinic acid, showed antioxidant activity in seven different in vitro tests. Furthermore, this antioxidant activity was also observed in vivo (*C. elegans*) and was shown to be independent of the presence of transcription factors. BT inhibits the accumulation of lipids in the model *C. elegans*, in diets with or without high amounts of glucose. Through RNAi, it was also observed that the transcription factor NHR-49 is involved in the mechanism of action of *B. trimera*. In addition, BT showed no toxicity against 3T3 cells and *C. elegans*. Together, the findings of this study support the idea that BT may be a promising alternative for the development of products for the prevention and treatment of obesity.

## Figures and Tables

**Table 1:** Evaluation of resistance to oxidative stress in nematodes treated with BT.

	Mean Survival (hours ± SEM)	*p* Value (Long Rank) Extract vs. Untreated	n
Control	24.73 ± 0.33 ^a^	< 0.0001	121 (3)
1.0 mg/mL	32.60 ± 0.49 ^b^	< 0.0001	127 (3)
5.0 mg/mL	32.70 ± 0.56 ^b^	< 0.0001	136 (3)

The letters a, b represent a significant difference between samples by the long-rank test (Mantel-Cox. n—total animals analyzed. The numbers between parameters indicate the number of independent analyses.

## Data Availability

All of the data is contained within the article.
